# Characterization of Eucalyptus, Chestnut and Heather Honeys from Portugal Using Multi-Parameter Analysis and Chemo-Calculus

**DOI:** 10.3390/foods7120194

**Published:** 2018-11-30

**Authors:** Ioannis K. Karabagias, Miguel Maia, Vassilios K. Karabagias, Ilias Gatzias, Anastasia V. Badeka

**Affiliations:** 1 Department of Chemistry, University of Ioannina, Laboratory of Food Chemistry, 45110 Ioannina, Greece; vkarambagias@gmail.com (V.K.K.); iliasgr1985@yahoo.gr (I.G.); abadeka@cc.uoi.gr (A.V.B.); 2 APISMAIA, Produtos & Serviços, Tlm: 962 889 512, Rua Almirante Reis, 91-A-2, 4490-463 Póvoa de Varzim, Portugal; info@apismaia.com

**Keywords:** Portuguese honey, characterization, palynological data, physico-chemical analysis, bio-functionality, volatiles, chemo-calculus

## Abstract

The present study was conducted to evaluate the quality and bio-functional properties of Portuguese honeys of different botanical and geographical origins. Quality parameter analyses included the determination of palynological (predominant, secondary, minor and isolated pollen percentage), physicochemical (°Brix, moisture content, pH, electrical conductivity, free acidity, total dissolved solids, salinity, vitamin C content and specific weight) including colour-metrics (CIELAB, Pfund and colour intensity determinations), along with volatile compounds identification using solid phase micro-extraction coupled to gas chromatography mass spectrometry. Bio-activity parameter analysis included the determination of in vitro antioxidant activity and total phenolic content using the 2,2-diphenyl-1-picryl-hydrazyl and Folin-Ciocalteu assays, respectively. Melissopalynological analysis showed that Portuguese honeys were classified as eucalyptus, chestnut and heather, recording significant variations (*p* < 0.05) among physicochemical, volatile and bio-activity parameter analyses according to both: botanical and geographical origin. Based on the multi-parameter analysis data Portuguese honeys could be characterized by a distinctive colour, a characteristic aroma, whereas conform to the European legislation relating to honey identity and quality. Specific attention should be given in the case of heather honey which showed the highest in vitro antioxidant activity and total phenolic content. Parameters that were also highly correlated using bivariate statistics.

## 1. Introduction

Honey, the sweet product of *Apis mellifera* honeybees, has been serving as an irritator of research the last decades, since numerous studies have been published related to its quality and authenticity. The EU Council directive relating to honey [1] has established some specific compositional criteria that should be followed for the determination of honey quality and declaration of origin. Therefore, its market distribution along with an attractive price is based on the latter’s identity. A prospective ideal identity demands the honey to possess a unique composition and properties. The contribution of scientific community to that good should be acknowledged since the botanical and geographical origin identification, composition of honey along with its properties has been exhaustively studied. Indeed, indicative studies have focused on melissopalynological analysis, sugar and moisture content determinations, colour determination, sensory analysis, volatile compounds identification, determination of honey minor components such as minerals, organic acids and polyphenols and so forth, alone or in combination, using chemo-calculus [2,3,4,5,6,7,8,9,10]. Therefore, it is important to characterize honey in depth, that is, from a multi-optional point of view. Chemo-calculus or more often chemometrics are the most decisive tools for an analyst to show and support any differences among the investigated matrix during the conduction of analyses [11].

In Portugal there are over 20 honey suppliers in Alentejo, Algarve, Central, Lisbon, Northern and other regions [12]. The annual honey production in Portugal is ca. 10,500 tons (10,451 in 2014) [13]. The area of Centre and Northeast Portugal is traditionally known for good quality honey production, especially *Eucalyptus globulus* (produced in North Coast), *Castanea sativ*a Mill., *Erica* spp. and some other honey types like honeydew and multifloral ones. Honey production represents an opportunity for the financial development of numerous regions in Portugal. Thus, the aim of the present work was to characterize eucalyptus, chestnut and heather honeys from six sub-regions of Portugal on the basis of palynological, physicochemical parameters including colour-metrics, volatile compounds and bio-activity parameter analyses using chemo-calculus on data obtained.

Even though there are previously published studies in the literature that highlight physicochemical composition and bio-functional properties, including that is, antioxidant and antimicrobial activity of Portuguese honeys [14,15,16,17,18,19,20,21], there is no study that combines palynological, physicochemical, volatile compounds and bio-activity parameter analysis data, to characterize Portuguese eucalyptus, chestnut and heather honeys, this contributing some new amendments to the state of the art.

## 2. Materials and Methods

### 2.1. Honey Samples

Seven honey samples were collected from different regions in Portugal during the harvesting seasons 2017 and 2018 due to the extent of natural limitations (Table 1). In particular, honey production was limited since a very dry spring, followed by a very hot and dry summer and even no autumn characterized the weather in Portugal. Therefore, the collection of nectar was very difficult for honeybees, resulting in a weak harvesting season for Portuguese apiculture. The sample codes and place of origin were: G1 (Braga, Esposende), G2 (Braga, Famalicão), G4 (Braga, Esposende), G5 (Viseu, Oliveira de Frades), G6 (Bragança, Vinhais), G7 (Braga, Vila Verde) and G8 (Coimbra, Vila Nova de Ceira). Samples were provided by local apiarists. All samples were stored at 4 ± 1 °C prior analysis.

### 2.2. Chemicals and Reagents

Gallic acid (3,4,5-trihydrobenzoic acid) anhydrous for synthesis was purchased from Merck (Darmstadt, Germany). Ethanol absolute for analysis, acetate buffer (CH_3_COONa·3H_2_O), Folin-Ciocalteu phenol reagent, sodium chloride (NaCl), sodium hydroxide (NaOH), sodium carbonate (Na_2_CO_3_), potassium iodide (KI) and iodine (I_2_) were purchased from Merck. Starch (from rice) was purchased from BIOTREK S.A.C.I. (Athens, Greece). 2,2-Diphenyl-1-picrylhydrazyl (DPPH) and acetic acid (CH_3_COOH) were purchased from Sigma-Aldrich (Darmstadt, Germany). Potassium chloride (0.1 M, 1413 μS/cm) used for the calibration of conductivity meter was purchased from Hanna (HI 7031, Hanna Instruments, Inc., Woonsocket, RI, USA).

### 2.3. Botanical Origin Identification

The botanical origin of Portuguese honey samples was confirmed using the melissopalynological analysis [22]. In particular, 10 g of each honey sample were diluted in 20 mL of distilled water and centrifuged at 4000 rpm for 5 min. The sediment of the solution was dried at 40 °C and mounted on glycerogelatin (Merck, Germany). The pollen grains were counted and identified in 1000× magnification using a VWR light microscope (VWR, model BL224T1-630-2672, Ponteranica, Bergamo, Italy). The determination of the botanical origin was based on the relative frequencies of nectariferous species. Pollen types from nectarless species were also recorded and counted separately. Only the pollen grain types with frequencies higher than 1% were considered.

### 2.4. Physicochemical Parameter Analyses

#### 2.4.1. Determination of Total Sugar and Moisture Contents

Total sugars (°Bx) and moisture content (%) were determined using a commercially available portable refractometer (ATC, Bellingham+ Stanley, UK). Prior the analysis of the honey samples, the refractometer was calibrated with a reference material (in our case extra virgin olive oil of 27% moisture). All honey samples were homogenized at room temperature and were directly deposited on the prism of the refractometer. The obtained refractive index in each sample was related to the water content of honey. The results of the measurements were expressed as a percentage (g/100 g). The refractometer used for the determination of sugars in honey samples was calibrated from the manufacturer in the range of 58–90% and in 12–27% for the determination of moisture. All measurements were performed in triplicate (*n* = 3) and results were expressed as average ± standard deviation values.

#### 2.4.2. Determination of pH

The pH of honey samples was measured in 10% (*w*/*v*) aqueous honey solutions, using a Delta OHM, model HD 3456.2, pH-meter (Padova, Italy) with a precision of 0.002 pH units. The instrument was calibrated with buffer solution (pH = 7.0 ± 0.002, Cat.22835-49) prior to measurements, which was obtained from HACH (Manchester, UK). Reported results are the average ± standard deviation values of three replicates (*n* = 3).

#### 2.4.3. Determination of Electrical Conductivity, Total Dissolved Solids and Salinity

The electrical conductivity of honey samples was measured in a 20% (*w*/*v*) honey solution in distilled water using a Delta OHM, model HD 3456.2, conductivity meter (Padova, Italy) at 25 °C. The probe was calibrated automatically resorting to the 1413 μS/cm conductivity standard solution (Hannah Instruments, Inc., Woonsocket, RI, USA). Temperature was measured by 4 wire Pt 100 and 2 wire Pt 1000 sensors by immersion. Given the fact that temperature was above 20 °C, the values were corrected by subtracting 3.2% of the value per °C [23]. Results were expressed as mS/cm and are the average ± standard deviation values of three replicates (*n* = 3).

Similarly, salinity and total dissolved solids of a 20% (*w*/*v*) aqueous honey solution in distilled water were measured at 20 °C using the aforementioned conductivity meter. Results were expressed as g/L and mg/L, respectively. All measurements were performed in triplicate (*n* = 3).

#### 2.4.4. Determination of Free Acidity

Free acidity was measured by dissolving 10 g of honey sample in 75 mL of carbon dioxide-free distilled water in a 250 mL beaker. The sample was then titrated with 0.1 N NaOH to pH 8.30 using phenolopthalein as the titration point indicator [23]. Results were expressed as milliequivalents/kg honey and are the average ± standard deviation values of three replicates (*n* = 3).

#### 2.4.5. Determination of Vitamin C Content

Vitamin C was determined by redox titration with iodine solution according to the method of College of Science, at the University of Canterbury [24]. The results reported are the average ± standard deviation values of three replicates (*n* = 3) and were expressed as mg of vitamin C per 100 g of honey considering the specific weight of each sample.

#### 2.4.6. Determination of Specific Weight

For the determination of specific weight (γ, g/mL) an aqueous solution of honey sample (10%, *w*/*v*) was prepared. Thereafter, 1 mL of this solution was inserted in a cuvette and weighted in a balance (Sartorius, BP 221 S, Malva, Greece) of four decimal points. Reported results are the average ± standard deviation values of three replicates (*n* = 3). All measurements were performed at 25 ± 1 °C.

#### 2.4.7. Colour-Metrics

##### Determination of CIELAB Colour Parameters

CIELAB colour parameters (L*, a*, b*), were determined according to CIE (Commission Internationale de l’ Eclairage) recommendations. The CIELAB system uses three parameters to evaluate colour in foodstuffs: (i) colour parameter L* corresponds to the degree of brightness; (ii) colour parameter a* (positive values) corresponds to the degree of redness and when a* shows negative values to the degree of greenness; and (iii) parameter b* corresponds to yellowness of colour (when positive) and to blueness of colour (when negative). What is remarkable is that the units within the L*, a*, b* system may provide equal perception of the colour difference to a human observer [21].

The aforementioned chromaticity coordinates of honey samples were measured using a Hunter Lab model DP-9000 optical sensor colorimeter (Hunter Associates Laboratory, Reston, VA, USA). The sample consisting of 10 g of honey in 75 mL distilled water was introduced in a glass petri dish and measurements were carried out by manual rotation of the sample in 45° of viewing aperture. Total difference of colour was calculated based on the following formula:

ΔE* = ((ΔL*)2 + (Δa*)2 + (Δb*)2)1/2 (1)

where ΔL*, Δa* and Δb* are the differences between the colour parameters of honey samples and the colour parameters of the white standard (YCIE = L* = 83.87, XCIE = a* = 81.82 and ZCIE = b* = 99.59) [25]. Reported values are the average ± standard deviation of five measurements (*n* = 5).

##### Determination of Colour Intensity: ABS_450_−ABS_720_

The colour intensity, defined as the net absorbance Abs450−Abs720, was an alternative methodology to evaluate colour of Portuguese honey samples. Honeys were diluted to a 50% proportion (*w*/*v*) with warm water (fixed temperature 45 °C) (AREX Heating Magnetic Stirrer, VTF Digital Thermoregulator, VELP Scientifica, Via Stazione, 16, 20,865 Usmate Velate Monza e Brianza, Italy) sonicated (Elma, Elmasonic model S 10H, Singen, Germany) for 5 min and then filtered using Whatman filters (Maidstone, UK) with a pore size of 0.45 μm, to remove any solid particles [26]. The spectrophotometer used was SHIMADJU, UV-1280 (Kyoto, Japan). Reported results are the average ± standard deviation values of three replicates (*n* = 3) and were expressed as mAU.

##### Determination of Colour According to Pfund Scale

Honey samples (50% aqueous honey solution, *w*/*v*) were heated to 50 °C to dissolve sugar crystals and the colour was determined spectrophotometrically at λ = 635 nm. The honeys were classified according to the Pfund scale after conversion of the absorbance values [27]:

Pfund (mm) = 38.70 + 371.39 × Abs(2)

##### In Vitro Determination of Antioxidant Activity of Portuguese Honeys

Preparation of DPPH Free Radical Standard Solution. The standard solution of [DPPH^•^] was prepared by dissolving 0.0044 g of the radical DPPH in 100 mL methanol. In that sense, the molarity of the obtained solution was 1.12 × 10^−4^ mol/L (M). The volumetric flask was wrapped in foil and stirred in a vortex apparatus. The solution obtained had a neutral pH (pH = 7.02), a deep purple colour and was left in the refrigerator for 2 h in order to stabilize [28].

Preparation of DPPH Free Radical Calibration Curve. A calibration curve of concentration versus absorbance of DPPH was prepared as follows: The 1.12 × 10^−4^ M (mol/L) solution of DPPH was diluted with the addition of methanol to cover the concentration range of 0–44 mg/L. The resulting solutions were vortexed, left in the dark (until measurements were made) and the absorbance was measured in the aforementioned UV/VIS Spectrometer at λmax of 517 nm. The calibration curve of absorbance (*y*) versus concentration (*x*) of [DPPH^•^] was expressed by the following equation:

*y* = 0.0174*x* − 0.0048; *R*^2^ = 0.9689 (3)

Determination of In Vitro Antioxidant Activity. All honey samples were dissolved in distilled water to obtain a concentration of 0.12 g/mL, that is a solution simulating the daily consumption of 30 g of honey dissolved in a glass of water (250 mL) (mother solution, *w*/*v*) [28].

Thereafter, volumes of 1.9 mL of methanol solution of [DPPH^•^] (0.044 mg/mL, 1.12 × 10^−4^ mol/L) and 1 mL of acetate buffer 100 mM (pH = 7.10) were placed in a cuvette and the absorbance of the [DPPH^•^] radical was measured at *t* = 0 (A_0_ = 0.8048).

Subsequently, 0.1 mL solution of each honey type was added to the above medium (final [DPPH^•^] concentration of 70.93 μmol/L) and the absorbance was measured every 30 min (regular time periods) until the value reached a plateau (steady state, At). The reaction was completed in 4 h. The absorbance of the reaction mixture was measured at 517 nm.

The [DPPH^•^] radical scavenging activity was calculated using the following equation:

%AA = (A_0_ − A_t_/A_0_) × 100 (4)

where A_0_ is the initial absorbance of the DPPH free radical standard solution and At is the absorbance of remaining [DPPH^•^] free radical after reaction with honey antioxidants, at steady state (t, plateau). The effective concentration of aqueous honey solutions that is the specific concentration that could decrease the [DPPH^•^] concentration by 50% was estimated based on the following formula:

EC_50_ (g/mL) = (Caq × 50%)/(%AA) (5)

where Caq: aqueous solution of honey added in the reaction medium (0.12 g/mL) and AA% the percentage inhibition of [DPPH^•^] after the addition of honey water soluble antioxidants.

Finally, methanol and acetate buffer (2:1, *v*/*v*) were used as the blank sample. Reported results are the average ± standard deviation values of three replicates (*n* = 3).

### 2.5. Determination of Total Phenolic Content

Total phenolic content of Portuguese honeys was determined in aqueous amounts of samples using the Folin-Ciocalteu colorimetric method [14]. In a 5 mL volumetric flask, 0.2 mL of the aforementioned aqueous honey solution (0.12 g/mL), followed by 2.5 mL of distilled water and 0.25 mL Folin-Ciocalteu reagent were properly added. Then, 0.5 mL of saturated Na_2_CO_3_ (30%, *w*/*v*) were also added after 3 min. Finally, the obtained solution was filled to the mark with distilled water (final volume 5 mL). This solution was left for 2 h in the dark (time starts when Folin-Ciocalteu reagent is added to the medium) at room temperature and the absorbance was measured at 760 nm in a UV/VIS Spectrophotometer (SHIMADJU, UV-1280, Japan). Prior absorbance measurements all solutions were filtered using Whatman filters (UK) with a pore size of 0.45 μm. For quantification purposes, a calibration curve was constructed of standard gallic acid using a range of concentrations between 195–6240 mg/L:

*y* = 0.0004*x* + 0.2041, *R*^2^ = 0.9954 (6)

Total phenolic content was expressed as mg of gallic acid equivalents per kg (mg GAE/kg) of honey, considering the specific weight of each honey sample. Reported results are the average ± standard deviation values of three replicates (*n* = 3).

### 2.6. Headspace Solid Phase Microextraction Coupled to Gas Chromatography/Mass Spectrometry (HS-SPME/GC-MS)

#### 2.6.1. Isolation of Volatile Compounds

The extraction of volatile compounds found in the headspace of honey samples was accomplished using a divinyl benzene/carboxen/polydimethylsiloxane (DVB/CAR/PDMS) fibre of 50/30 μm purchased by Supelco (Bellefonte, PA, USA). Before analysis of samples the fibre was conditioned according to the manufacturer’s recommendations and was cleaned daily using the method of “clean” program. In particular, the injector and MS-transfer line were maintained at 260 °C and 270 °C, respectively, whereas during the “cleaning” of the fibre oven temperature was held at 80 °C for 0 min and then was increased to 270 °C at 10 °C/min (2 min hold). A split ratio of 10:1 was used.

For honey sample analysis the following conditions were followed: 15 min equilibration time, 30 min sampling time, 4 mL sample volume, addition of salt and 45 °C water bath temperature. The samples (2 g honey in 2 mL of distilled water, plus 0.20 g NaCl (Merck, Darmstadt, Germany) plus 20 μL of internal standard (benzophenone, 100 μg/mL, Sigma Aldrich, St. Louis, MO, USA), were then placed in 15 mL screw-cap vials (a magnetic stirrer was also placed inside the vials) equipped with PTFE/silicone septa. The vials were maintained at 45 °C in a water bath under continuous stirring at 600 rpm during the entire headspace extraction [10].

#### 2.6.2. Gas Chromatography-Mass Spectrometry Unit and Analysis Conditions

The GC unit used in the study for the gas chromatography/mass spectrometry analysis of honey samples was an Agilent 7890A model coupled to a MS detector (Agilent 5975, Santa Clara, CA, USA). The capillary column used in the analysis was DB-5MS (cross linked 5% PH ME siloxane) (60 m × 320 μm i.d., ×1 μm film thickness). Helium served as the carrier gas (purity 99.999%), at a flow rate of 1.5 mL/min. The injector and MS-transfer line were maintained at 250 °C and 270 °C, whereas during the analysis oven temperature was held at 40 °C for 3 min and then was increased to 260 °C at 8 °C/min (6 min hold). Electron impact mass spectra were recorded at 50–550 mass range and the ionization energy was 70 eV, whereas a split ratio of 1:2 was used to introduce the appropriate amount of sample on column. For the analysis of honey samples of different botanical origin, blank runs were carried out to avoid any source of contamination

Identification and semi-quantification of honey volatile compounds assuming a response factor equal to 1.

The identification of volatile compounds was achieved using the Wiley 7, NIST 2005 mass spectral library. For the calculation of Kovats indices, a mixture of n-alkanes (C8–C20) supplied by Supelco (Bellefonte, PA, USA) was dissolved in n-hexane and the retention time of standards was determined according to the temperature-programmed run discussed above. Volatile compounds having ≥80% similarity with Wiley library were tentatively identified using GC-MS spectra. The method of identification was based on the combination of MS data found in Wiley 7 NIST 2005 mass spectral library and data of Kovats index values that were determined for each volatile compound and then compared with those included in the Wiley MS library.

For the semi-quantification analysis the internal standard method was used. Data were expressed as concentration (Canalyte, mg/kg) based on the ratio of peak areas of the isolated volatile metabolites to that of the internal’s standard, multiplied by the final concentration of the internal standard (1 mg/L), assuming a response factor (RF) equal to 1 for all the compounds [10]. Data were then expressed as μg/kg. Volatile compounds identified only in replicated samples were used in the study.

### 2.7. Statistical Analysis

Data of honey samples were subjected to analysis of variance (ANOVA) in order to show any statistical differences (*p* < 0.05) according to botanical origin (eucalyptus, chestnut and heather). Furthermore, in order to evaluate the effect of geographical origin on physicochemical and bioactivity parameter values, especially in the case of eucalyptus and chestnut honeys, in which samples originated from different regions, T-test was applied (*p* < 0.05). Correlations were obtained by Pearson’s correlation coefficient (*r*), at the confidence level *p* < 0.05. All data processing was performed using the SPSS v.20.0 statistics software (SPSS Inc., Chicago, IL, USA).

## 3. Results and Discussion

### 3.1. Botanical Origin Identification of Portuguese Honey Samples

Melissopalynological analysis showed the presence of *Eucalyptus* spp. (samples G1, G2, G4 and G7), *Castanea sativa* Mill. (samples G5 and G6) and *Erica* spp. (sample G8) pollen grains at a percentage of >45%. Therefore, these pollen grains were the dominant ones. The presence of secondary (16–45%), minor (3–15%) and identified (<3%) pollen was also confirmed (Table 1). Based on the dominant pollen grains percentage, the botanical origin of Portuguese honey samples was: Eucalyptus, Chestnut and Heather.

The *Eucalyptus* spp. pollen grains among honey samples analysed ranged between 73–82%. In a previous work dealing with eucalyptus Portuguese honeys produced in the Entre-Douro e Minho region Feás et al. [17] reported a pollen grain percentage of *Eucalyptus* spp. pollen grains ranging between 44–79%, with an average value of ca. 58%. Estevinho et al. [18] in a study carried out on commercial honeys available in the Portuguese market reported that eucalyptus honey samples showed a pollen grain percentage of *Eucalyptus* spp. between ca. 50–71%.

In another study involving heather honey (*Erica* spp.) from six districts in Portugal (Aveiro, Graga, Porto, Viseu, Viana do Castelo, Vila real) Pires et al. [29] reported that the number of *Erica* spp. pollen grains per sample varied between 45% and 71%, with an average ± standard deviation value equal to 56 ± 9%. Heather honey from Trás-Os-Montes region in Portugal showed an average value of *Erica* spp. pollen grains ca. 60 ± 1% [19]. The contribution of *Castanea sativa* Mill. pollen grains among *Erica* spp. honeys was also reported by the authors, in agreement with the results of the present study (Table 1). At this point, it should be stressed that the most common *Erica* species in the Iberian Peninsula are *E. arborea*, *E. australis*, *E. umbellata* and others [30]. However, during the melissopalynological analysis these species were not identified. In that sense, the general term “*Erica spp*.”, involving other *Erica* species [30], was used to characterize the heather honey sample. Therefore, the geographical origin of Portuguese honeys may have also a strong impact on the total contribution (% percentage) of specific pollen grains. Honeys were then grouped according to botanical and geographical origin for the physicochemical and bio-activity parameter analyses.

### 3.2. Physicochemical Parameter Values of Portuguese Honey Samples

Sugars and moisture comprise the major components of honey. Total sugar content (°Brix) recorded the higher value in chestnut honey from Bragança, in Vinhais County. In all honey samples analysed total sugars recorded values higher than 80% (g/100 g). There were observed significant differences (*p* < 0.05) in total sugar content among eucalyptus and chestnut honeys of different geographical origin (Table 2). The present results agree with those of Silva et al. [15] in a study carried out on eucalyptus and heather honeys harvested in Luso province (centre region of Portugal).

In addition, significant differences (*p* < 0.05) were observed among honey types of different botanical and geographical origin in the moisture content. Moisture content of Portuguese honeys ranged between 15.23 ± 0.15 to 17.93 ± 0.06. The lowest value was recorded for chestnut honey from Bragança region and the highest value was recorded for eucalyptus honey from Esposende region. In total, moisture content values are in accordance with the EU council directive relating to honey [1], in which the value of 20% (g/100 g) has been defined as the upper moisture limit for marketed honeys. Of course some exceptions may occur in honey types such as *Erica* spp. (*Calluna*) in which the moisture upper limit may be 23%. From a scientific point of view, the determination of moisture is related to honey preservation and storage, as high water content can lead to the growth of moulds, that may alter a product’s flavour and shelf-life [16]. However, the differences observed between honey samples may be due to environmental conditions, harvesting year and the degree of honey maturity reached in the beehive [31]. These moisture content values agree with previous studies on Portuguese honeys [15,17,18,19,29,32].

The pH values ranged from 3.62 ± 0.01 to 4.42 ± 0.01, with significant differences (*p* < 0.05) between samples (Table 2). Briefly, the lower pH value (effective acidity) was recorded for eucalyptus honey from Esposende region, whereas the higher pH value was recorded for chestnut honey from Bragança region. Even though there is not a specified pH value for honey the determination of this parameter is of significant importance as it may influence honey texture, stability and shelf life [32]. Low pH values inhibit the presence and growth of microorganisms. Such pH values are consistent with some studies on Portuguese honeys [16,17,18,19,29].

Electrical conductivity of honey is owed to minerals and trace elements, small amounts of proteins or any other charged molecule released in the aqueous form of a honey solution. It is a conventional physicochemical parameter that it has been correlated with the botanical origin of honey and it is used for honey botanical origin identification in combination with melissopalynological analysis data. The eucalyptus honeys showed the lower electrical conductivity values, whereas chestnut honeys the highest (Table 2). The electrical conductivity values of eucalyptus honey samples analysed in the present study are in conformity with previous studies in the literature involving eucalyptus honeys [17]. The electrical conductivity value of heather honey is in excellent agreement with those of a previous study on Portuguese heather honeys [29]. According to the EU Council directive [1] the maximum limit value of electrical conductivity for blossom honeys is 0.80 mS/cm, while honeydew honeys have electrical conductivity values ≥0.80 mS/cm.

Total dissolved solids reflect all inorganic and organic molecules that are present in honey in molecular, ionized or micro-granular (colloidal solution) suspended forms [33]. TDS recorded higher values (mg/L) in chestnut honeys followed by those of heather and eucalyptus honeys (Table 2). What is remarkable is that the observed differences were significant (*p* < 0.05) according to Portuguese honey botanical and geographical origin. On the other hand, salinity is a measure of the saltiness or dissolved salt content in an aqueous medium. For instance, chestnut honeys recorded the highest salinity values (g/L) followed by heather and eucalyptus honeys.

To the best of our knowledge, data on TDS and salinity contents have never been reported before for Portuguese honeys. Available data in the literature originate from Algerian, Greek, Cypriot and Egyptian floral honeys [33,34], in which TDS and salinity contents values recorded fluctuations according to honey botanical and geographical origin, in agreement with the results of the present study.

Free acidity of honey is owed to its organic acid content in terms of gluconic, formic, malic, succinic, oxalic, butyric, citric, 2,3-dihydroxybutanedioic, pyroglutamic, lactic, benzoic, maleic, isobutyric, pyruvic, α-ketoglutaric and glycolic acids. Despite the fact that organic acids are found in minor proportions in honey (0.17–1.17 g/100 g), these play an important role in the developed aroma and flavour of honey, since the majority of the aforementioned acids are present in honey in the form of volatile esters. On the other hand, storage temperature, processing conditions and the nectar source may influence organic acid content of honey. In addition, an increased acidity may serve as an indicator of honey fermentation and transformation of alcohol into organic acid [35]. The free acidity values for the three Portuguese honey types studied were below the limit of 50 meq/kg set by the European directive relating to honey [1]. The highest free acidity values (meq/kg) were recorded for heather honeys. In a previous work carried out on Portuguese heather honeys free acidity ranged between 10.5–38.1 meq/kg [15]. Gomes et al. [32] reported average ± standard deviation values of free acidity equal to 27.00 ± 5.00 for *Eucalyptus* spp. honeys purchased from local markets in Portugal.

Vitamin C recorded the highest value (mg/100 g) in heather honey. Even though honey is not a rich source of vitamin C compared to fruits like orange, lemon and so forth, hence, vitamin C contributes to the total antioxidant activity of honey and more specifically to that of the water soluble antioxidants that are present [28]. Previous studies in the literature on Portuguese honeys (rosemary, viper’s bugloss, heather) have reported values in the range of ca. 14.00–14.58 mg/100 g. The highest value in vitamin C was recorded for heather honey from the region of Portela in Northeast Portugal [14]. What is in full agreement with the results of the present study is that vitamin C was affected by honey botanical origin.

### 3.3. Colour-Metrics of Portuguese Honey Samples

In Table 3 are given the values of chromaticity coordinates, expressed as average ± standard deviation. The results obtained show that the lightest honey was that of eucalyptus (sample G2), whereas the darker was that of heather honey (sample G8). In relation to the dark colour of heather honey, this is probably due to the contribution of chestnut in its pollen spectrum. Even though one heather honey sample was used in the study, this preliminary finding in nature agrees with the results of Soares et al. [21], involving heather honey produced in North and Centre Portugal (Vila Real, Bragança, Chaves, Boticas, Lousã, Penamacor, Vila Nova de Foz Côa and Viseu). In addition, greenish (negative a* values) and yellow components (positive b* values) (pigments) were present in all honey samples analysed in conformity with the aforementioned work [21]. What is important is that these components varied significantly (*p* < 0.05) according to honey geographical origin. Comparing the chromaticity values of eucalyptus, chestnut and heather honeys, only L* and b* values varied significantly (*p* < 0.05) according to honey botanical origin.

The difference or distance between colour parameters (ΔΕ* or ΔΕab*) is a metric of interest in colour science. The determination is normally based in the Euclidean distance and it allows a “quantified” theory of an aspect that could only be described with single values. It is a critical measure in the colour science and may define how colour travels in an independent multi-dimensional space. The ΔE* determination might be of interest since the human eye is more sensitive to certain colours than others. Results showed that ΔΕ* values recorded significant differences (*p* < 0.05) among honey samples of different botanical origin. The highest values were recorded for eucalyptus honeys followed by chestnut and heather honeys.

The U.S. Department of Agriculture has classified honey into seven colour categories according to its colour: water white, extra white, white, extra light amber, light amber, amber and dark amber. The Pfund colour scale may provide an accurate, inexpensive and convenient methodology for measuring colour intensity of honey as a distance (in mm) in the chromatic space. Respective values (in mm) are: <9 for water white, 9–17 for extra white, 18–34 for white, 35–50 extra light amber, light amber 51–85, amber 86–114 and dark amber > 114 [36]. Portuguese honey samples analysed showed a diverse colour according to Pfund scale. More specifically, eucalyptus honeys’ colour could be characterized as extra light amber to light amber; chestnut honeys’ colour could be characterized as light amber to amber; whereas heather honey’s colour could be characterized as dark amber (Table 2). What is worthy of mentioning is that colour of honeys analysed, according to Pfund scale, varied significantly (*p* < 0.05) with respect to botanical and geographical origin.

Ferreira et al. [14] characterized chestnut honey colour from Northeast Portugal as dark according to Pfund scale results. In the same line of reasoning, Gomes et al. [32] reported that eucalyptus honey from local markets in Portugal had an amber colour. Argentinean eucalyptus honeys possessed an extra light–amber to amber colour [37].

Colour intensity, defined as the difference in the absorbance at 450–720 nm, may give information about the presence of pigments (polyphenols, carotenoids, etc.) in honey. It has been reported in the literature that dark coloured honeys usually have higher colour intensity values [26,28,33]. Indeed, heather honey from Coimbra recorded the highest colour intensity values followed by chestnut and eucalyptus honeys. The differences among honey samples of different botanical and geographical origin were also significant (*p* < 0.05).

### 3.4. Bio-Activity Parameter Values of Portuguese Honey Samples

Spectrometric assays like DPPH and Folin-Ciocalteu may be routinely used for the in vitro characterization of honey bio-functional properties. The DPPH assay evaluates the antioxidant activity of a sample. The free radical can be neutralized either by direct reduction (via electron transfer) or by radical quenching (via H atom transfer) [21]. It has been reported previously in the literature that dark coloured honeys usually have a higher in vitro antioxidant activity [26,28,38]. Among Portuguese honey samples analysed heather honey (darkest honey) showed the higher in vitro antioxidant activity followed by chestnut and eucalyptus honeys (Table 2). The higher in vitro antioxidant activity of heather honey, compared to other types, has been previously reported [14,21] in agreement with the results of the present study.

In general Portuguese honey samples showed a higher in vitro antioxidant activity compared to Polish lime, nectar-honeydew, rape, honeydew, acacia and buckwheat honeys [38]. Greek pine and fir honeys recorded in vitro antioxidant activity values within the range of eucalyptus honeys analysed in the present work [28].

Another important issue to discuss is that the antioxidant activity not only depends on the botanical origin of honey but also on the geographical origin (Table 2). Parameters such as plants’ defence against several environmental factors including climatic conditions, ultraviolet radiation, temperature, water stress or mineral nutrient availability may imply significant parameters that affect honey in vitro antioxidant activity [21,28].

The method of Folin-Ciocalteu is largely used to evaluate total phenolic content despite the arising interferences of this assay since the reagent mixture (phosphotungstic acid and phosphomolybdic acid) also reacts with other non-phenolic reducing compounds (ascorbic acid, amino acids, etc.) and affects absorbance measurements, therefore leading to an overestimation of the phenolic content [14]. However, it is a simple and effective procedure to estimate total phenolic content of foodstuffs with bio-functional properties.

The lowest TPC content was recorded for eucalyptus honey from Esposende region (Braga) (ca. 422 mg GAE/kg), while the highest phenolic content (ca. 1418 mg GAE/kg) was found in chestnut honey from Vinhais region (Braganca), followed by heather honey (ca. 1380 mg GAE/kg) from Vila Nova de Ceira region (Coimbra) being much higher than the results reported for total phenolic content of heather honey (ca. 500 mg GAE/kg) from Boticas region in Portugal [21]. Some other researchers have also reported a trend for heather honeys to show a higher total phenolic content [14,20,39]. Chestnut honey from Croatia [40] recorded a total phenolic content of ca. 430 mg GAE/kg. Such values are much lower compared to the results obtained for Portuguese chestnut honeys.

But what is the main reason for such fluctuations? At first, the analytical instrumentation and methodology have definitely a significant effect on the quantification of such compounds. At the same time and in a greater extent, climatic and soil conditions may fully describe such phenomena. Bees collect pollen from different plants grown in different regions. Pollen is a good source of numerous phytochemicals. In that sense, polyphenols and other bio-functional compounds are transferred to honey through pollen, which can justify the observed fluctuations in TPC content among honey samples of different botanical and geographical origins [21].

Finally, the antimicrobial activity of honey against a wide range of microbial contaminants (aerobic mesophilic bacteria, moulds and yeasts, faecal coliforms, sulphite-reducing clostridia, *Salmonella*, etc.) along with its wound healing boosters should be considered in its overall bio-functionality [19,41]. Even though this data was not collected, the physicochemical and bio-functional activity parameters were measured (pH, acidity, moisture, antioxidant activity, total phenolic content, volatile compounds), creating the basis for a “promising” antimicrobial activity and wound healing properties of Portuguese honeys. Indeed, pH, acidity, hydrogen peroxide, phenolic and volatile compounds have been reported to contribute to the biological activity of honey [32].

### 3.5. Correlations between Measured Physico-Chemical, Palynological and Bioactivity Parameters of Portuguese Honeys Using Pearson’s Bivariate Statistics

Despite the limited honey samples analysed, there were collected some important data regarding physico-chemical, palynological and bioactivity parameter values correlations that could be indicative of the specific Portuguese honeys analysed. The bivariate Pearson’s correlation (*r*) was used at the confidence level *p* < 0.05. In particular, a slight positive but insignificant correlation (*r* = 0.108, *p* = 0.819) was obtained for pH and vitamin C content values. This was also the case for free acidity and pH values of Portuguese honeys (*r* = 0.276, *p* = 0.549). Total colour difference (ΔΕ*) of honey samples using the CIELAB system was negatively but significantly, correlated with the Pfund scale colour determination (*r* = −0.881, *p* = 0.009). To our knowledge, there are limited studies upon such correlations.

Regarding the correlation between pollen grain percentages and colour parameters some important findings were also observed. In the case of eucalyptus honeys, pollen grains were negatively correlated with ΔE* values (*r* = −0.95, *p* = 0.050); slightly positively correlated with Pfund scale measurements (*r* = 0.25, *p* = 0.749); and positively correlated with colour intensity measurements (*r* = 0.70, *p* = 0.295). For the chestnut honeys respective correlations were *r* = −1 (*p* = 0.01*)*, *r* = 1 (*p* = 0.01), and *r* = 1 (*p* = 0.01). As it can be observed the correlations obtained between colour parameter analyses and pollen grain percentages for chestnut honeys were more promising.

Finally, for bio-activity parameter values more positive results were obtained. Total phenolic content was strongly correlated with the in vitro antioxidant activity (*r* = 0.901, *p* = 0.006). In addition, there was also a strong positive correlation between total phenolic content and colour intensity values (*r* = 0.836, *p* = 0.019). Such findings are in accordance with previous works in the literature upon this theory [14,21,26,41,42,43]. However, for the first time in the literature there has been carried out an effort to correlate Pfund colour scale measurements with those of colour intensity. Results, hence, showed that there was an almost perfect Pearson’s correlation between this two measures (*r* = 0.975, *p* = 0.000).

### 3.6. Volatile Compounds of Portuguese Honeys

**Figure 1:**
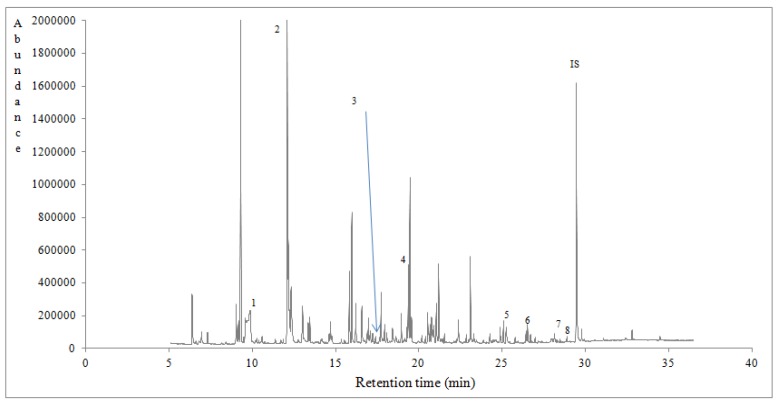
A typical gas chromatogram of eucalyptus honey from the region of Viseu, Oliveira de Frades in Portugal. 1: 2-Butanone. 2: Octane. 3: alpha-Phellandrene. 4: Linalool. 5: 2-Buten-1-one. 6: 2,6-bis-(1,1-trimethyl)-2,5-cyclohexadiene-1,4-dione. 7: alpha-Calacarene. 8: Byciclo [5.3.0] decapentaene. IS: Internal standard.

**Figure 2:**
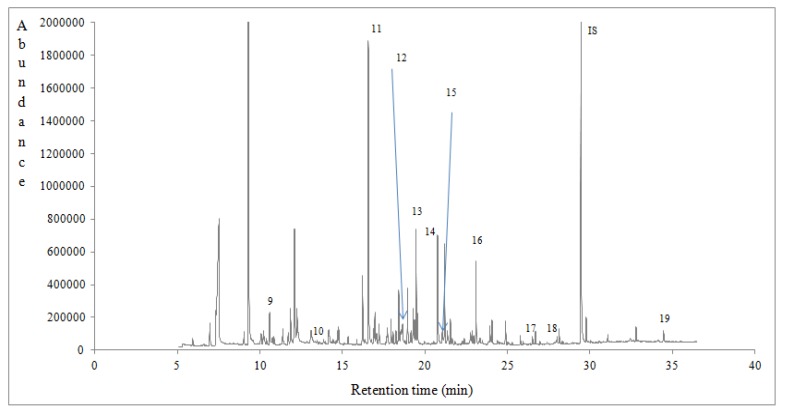
A typical gas chromatogram of chestnut honey from the region of Braganca, Vinhais, in Portugal. 9: 2-methyl-2-Butenal. 10: 3-methyl-butanoic acid ethyl ester. 11: Benzaldehyde. 12: 2-hydroxy-Benzaldehyde. 13: Nonanal. 14: 1-Nonanol. 15: Benzoic acid ethyl. 16: Nonanoic acid ethyl ester. 17: 2,6-bis-(1,1-trimethyl)-2,5-cyclohexadiene-1,4-dione. 18: Dodecanoic acid ethyl ester. 19: Hexadecanoic acid ethyl ester. IS: Internal standard.

**Figure 3:**
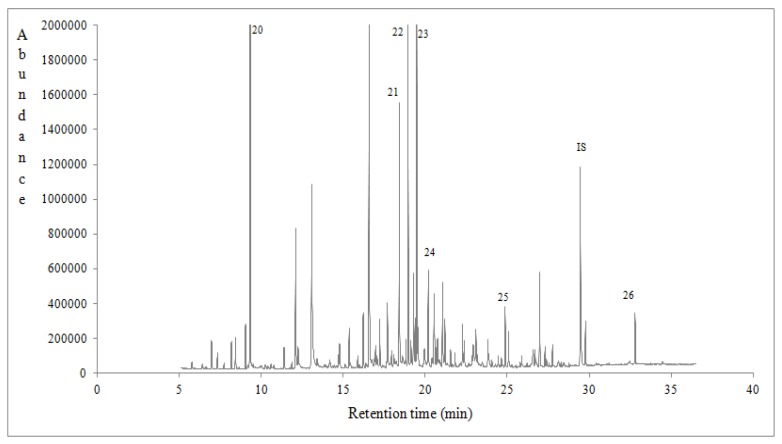
A typical gas chromatogram of heather honey from the region of Coimbra, Vila Nova de Ceira, in Portugal. 20: Heptane. 21: Benzeneacetaldehyde. 22: cis-Linalool oxide. 23: Hotrienol. 24: alpha-Isophorone 25: 1,2-dihydro-1,1,6-trimethyl-Naphthalene. 26: Eicosane. IS: Internal standard.

Ninety eight volatile compounds of different class were tentatively identified and semi-quantified using a response factor equal to 1 for all the compounds [8,34] (Table 4). In Table 4 are listed the compounds that were identified with a qualification MS value of ≥83. The volatile compounds of eucalyptus, chestnut and heather honeys could be classified as alcohols, aldehydes, benzene derivatives, hydrocarbons, esters, furan derivatives, ketones, norisoprenoids, phenolic volatiles, terpenoids, sulphur volatiles and so forth. What is remarkable is that volatiles were significantly affected (*p* < 0.05) by honey botanical (Table 4) and geographical origin (data not shown). Total volatile compounds semi quantitative data (μg/kg) of Portuguese honeys followed the sequence: heather (21,257.17 ± 1051.56) > eucalyptus (8488.22 ± 3180.80) > chestnut (8448.52 ± 1626.72). Many of these compounds comprise some typical volatile markers of monofloral honeys harvested in different parts of the world [8,34,44,45]. Typical gas chromatograms of eucalyptus, chestnut and heather honeys are shown in Figure 1, Figure 2 and Figure 3, indicating with numbers some characteristic volatile markers according to honey botanical origin.

Castro-Vázquez et al. [44] using solid phase microextraction followed by gas chromatography coupled to mass spectrometry reported the presence of 35 volatile compounds in Spanish eucalyptus honeys. Volatiles such as isoborneol (or borneol), nonanal, benzaldehyde, benzeneacetaldehyde (or phenylacetaldehyde), 4-oxoisophorone (or 4-ketoisophorone) were identified in considerable amounts (μg/kg), in agreement with present results involving Portuguese eucalyptus honeys.

Pontes et al. [45] using headspace solid phase microextraction coupled to gas chromatography quadrupole mass spectrometry reported the presence of 110 volatile compounds in wildflower, eucalyptus, hissed and rosemary honeys from four different regions of Madeira Island. Among the aforementioned volatile compounds, linalool, trans-linalool oxide, 4-ketoisophorone, benzaldehyde, benzeneacetaldehyde, decanal, naphthalene and so forth, were identified in Eucalyptus spp. honeys, in conformity with present results.

Karabagias et al. [34] using solid phase microextraction coupled to gas chromatography mass spectrometry reported that 53 volatile compounds dominated the headspace aroma of Greek chestnut honeys. Numerous of the aforementioned compounds were also identified in Portuguese honeys of the present work. However, specific attention should be given to volatile compounds such as heptane, 2,4,5-trimethyl-1,3-dioxolane, 2-methyl-2-butenal, 3-methyl-butanoic acid ethyl ester, benzeneacetic acid ethyl ester,1-(2-aminophenyl)-ethanone,1-(6-methyl-3-pyridinyl)-ethanone,2,6-di-butyl-2,5-cyclohexadiene-1,4-dione, eucalyptol, alloaromadendrene, hexadecanoic acid ethyl ester, which were identified only in Portuguese chestnut honeys and not in the Greek ones. On the other hand, the volatiles 2-heptanone, 1-octanol, undecanal, dodecanal, 1-methoxy-4-propyl-benzene, were identified only in Greek chestnut honeys [34] and not to the Portuguese ones, indicating the impact of honey geographical origin on its volatile composition and the overall aroma character. Regarding Portuguese honeys, apart from the common volatile compounds found in other monofloral honeys, such as benzaldehyde, octanal, 2-ethyl-1-hexanol and so forth [7,8,10,34,43,44], alloaromadendrene and a-calacorene has been reported to possess a woody odour, whereas azulene is still unknown what its characteristic flavour is [46]. What is also worthy of mentioning, is the high amounts (μg/kg) of heptane, benzaldehyde, benzeneacetaldehyde, cis-linalool oxide, hotrienol found in heather honey. Therefore, the amount and synergistic action of the isolated volatiles reveals a unique aroma for Portuguese honeys. To our knowledge, there are limited studies in the literature that enhance the volatile profile of Portuguese honeys [45] and especially that of heather honey.

## 4. Conclusions

Results of the present study showed that both botanical and geographical origin of Portuguese honeys had a significant impact (*p* < 0.05) on physicochemical and bio-functional activity parameters. Colour-metrics revealed that colour of Portuguese eucalyptus, chestnut and heather honeys could be defined as light-amber to amber with yellow and green components (phytochemicals). Heather honey showed the higher in vitro antioxidant activity, total phenolic and vitamin C contents. The aroma of honeys was affected by botanical and geographical origin and was the outcome of the synergistic action of plenty volatile compounds. In addition, some important correlations among physico-chemical, palynological and bio-functional activity parameters were obtained, enhancing further the identity of Portuguese honeys. Present data support the literature on Portuguese honeys and may be used by different agencies in studies/research related to characterization, authentication and adulteration control of Portuguese honey.

## Figures and Tables

**Table 1:** Sample coding, harvesting year and melissopalynological data of Portuguese honey samples analysed in the present study.

Sample Code	Harvesting Year	Predominant Pollen (>45%)	Secondary Pollen (16–45%)	Minor Pollen (3–15%)	Identified Pollen (<3%)
G1	2018	*Eucalyptus* spp.: 73%	*Raphanus raphanistrum*: 17%	*Acacia* spp.: 3%, *Salix* spp.: 3%	*Sinapsis arvensis*: 2%, *Trifolium* spp.: 1%
G2	2018	*Eucalyptus* spp.: 82%	-	*Raphanus raphanistrum:* 11%, *Echium plantagineum*: 3%	*Salix* spp.: 1%, *Rubus* spp.: 1%
G4	2018	*Eucalyptus* spp.: 78%	-	*Raphanus raphanistrum*: 10%, *Salix* spp.: 6%, *Acacia* spp.: 3%	*Castanea sativa* Mill.: 1%, *Echium plantagineum*: 1%
G5	2017	*Castanea sativa* Mill.: 85%	-	*Rubus* spp.: 11%	*Lavandula stoechas*: 1%, *Crataegus monogyn*a: 1%
G6	2017	*Castanea sativa* Mill.: 82%	-	*Eucalyptus* spp.:6%, *Rubus* spp.:6%, *Echium plantagineum*: 3%	*Raphanus raphanistrum*: 1%, *Erica* spp.: 1%, *Cytisus* spp.: 1%
G7	2017	*Eucalyptus* spp.: 79%	-	*Erica* spp.:7%, *Rubus* spp.: 5%, *Castanea sativa* Mill.: 4%	*Raphanus raphanistrum*:2%, *Salix* spp. :2%, *Solanum* sp.: 1%
G8	2018	*Erica* spp.: 46%	*Castanea sativa* Mill.: 30%	*Eucalyptus* spp.: 10%, *Lotus creticus*: 3%, *Rubus* spp.: 3%, *Acacia* spp.: 3%	*Trifolium* spp.: 2%, *Cytisus* spp.: 1%, *Sesamoides* spp.: 1%, *Frangula alnu*s: 1%, *Salix* spp.: 1%

**Table 2:** Physicochemical parameters of Portuguese honeys according to botanical and geographical origin.

Botanical Origin	District	County	Total Sugars (Brix)	Moisture (g/100 g)	pH	EC (mS/cm)	TDS (mg/L)	Salinity (g/L)	Free Acidity (meq/kg)	Vitamin C (mg/100 g)	Specific Weight (g/mL)
Eucalyptus.	Braga	Esposende	81.13 ± 0.25 ^a^	16.85 ± 0.10 ^g^	3.73 ± 0.01 ^m^	0.33 ± 0.00 ^s^	197.7 ± 0.15 ^y^	0.19 ± 0.00 ^af^	15.50 ± 0.58 ^am^	16.07 ± 0.93 ^as^	1.017 ± 0.01 ^az^
Eucalyptus.	Braga	Famalicão	81.37 ± 0.12 ^a^	17.03 ± 0.06 ^g^	3.72 ± 0.01 ^m^	0.35 ± 0.00 ^t^	213 ± 2.00 ^z^	0.21 ± 0.00 ^ag^	17.33 ± 0.60 ^an^	14.09 ± 0.00 ^at^	1.011 ± 0.01 ^aaa^
Eucalyptus.	Braga	Esposende	80.30 ± 0.26 ^b^	17.93 ± 0.06 ^h^	3.62 ± 0.01 ^n^	0.45 ± 0.00 ^u^	272 ± 1.00 ^aa^	0.27 ± 0.00 ^ah^	14.33 ± 0.58 ^ao^	10.57 ± 0.00 ^au^	1.019 ± 0.01 ^aab^
Eucalyptus.	Viseu	Oliveira de Frades	82.60 ± 0.00 ^c^	15.87 ± 0.00 ^i^	3.98 ± 0.01 ^o^	0.42 ± 0.00 ^v^	295.33 ± 0.58 ^ab^	0.29 ± 0.00 ^ai^	16.67 ± 0.58 ^ap^	11.01 ± 0.00 ^av^	1.031 ± 0.01 ^aac^
Chestnut.	Bragança	Vinhais	83.07 ± 0.06 ^d^	15.23 ± 0.15 ^j^	4.42 ± 0.01 ^p^	1.14 ± 0.26 ^w^	679.33 ± 1.53 ^ac^	0.68 ± 0.00 ^aj^	19.67 ± 0.58 ^aq^	12.33 ± 0.00 ^aw^	1.049 ± 0.01 ^aad^
Chestnut.	Braga	Vila Verde	81.50 ± 0.00 ^e^	16.87 ± 0.06 ^k^	4.35 ± 0.01 ^q^	0.98 ± 0.001 ^w^	582 ± 1.0 ^ad^	0.58 ± 0.00 ^ak^	19.67 ± 0.58 ^aq^	15.85 ± 1.25 ^ax^	1.045 ± 0.01 ^aae^
Heather	Coimbra	Vila Nova de Ceira	82.03 ± 0.06 ^f^	16.47 ± 0.06 ^l^	4.03 ± 0.01 ^r^	0.71 ± 0.00 ^x^	427 ± 2.65 ^ae^	0.43 ± 0.00 ^al^	30.33 ± 1.53 ^ar^	35.66 ± 0.00 ^ay^	1.036 ± 0.01 ^aaf^

Different letters in each column represent statistically significant differences at confidence level *p* < 0.05. Results reported are the average ± standard deviation values of three replicates (*n* = 3).

**Table 3:** Colour-metrics and bioactivity parameter values of Portuguese honeys according to botanical and geographical origin.

Botanical Origin	District	County	L*	a*	b*	ΔE^*^	Pfund (mm)	Colour Intensity (mAU)	%AA	TPC (mg/kg)	EC_50_ (g/mL)
Eucalyptus	Braga	Esposende	77.09 ± 0.16 ^a^	−3.81 ± 0.16 ^g^	6.66 ± 0.18 ^n^	126.55 ± 0.29 ^u^	66.3 ± 0.08 ^aa^	433.7 ± 0.32 ^ai^	46.86 ± 0.04 ^an^	422 ± 0.58 ^au^	0.13 ± 0.00 ^aab^
Eucalyptus	Braga	Famalicão	77.48 ± 0.09 ^b^	−3.50 ± 0.18 ^h^	7.13 ± 0.22 ^o^	125.97 ± 0.30 ^v^	43.36 ± 0.06 ^ab^	432.9 ± 0.10 ^ai^	42.97 ± 0.02 ^ao^	549 ± 0.00 ^av^	0.14 ± 0.00 ^aac^
Eucalyptus	Braga	Esposende	77.14 ± 0.11 ^a^	−2.42 ± 0.29 ^i^	5.76 ± 0.20 ^p^	126.28 ± 0.37 ^u^	60.60 ± 0.04 ^ac^	433.7 ± 0.32 ^ai^	52.80 ± 0.01 ^ap^	449 ± 0.22 ^aw^	0.11 ± 0.00 ^aad^
Eucalyptus	Viseu	Oliveira de Frades	76.66 ± 0.05 ^c^	−4.21 ± 0.35 ^j^	8.71 ± 0.14 ^q^	125.35 ± 0.36 ^w^	88.40 ± 0.12 ^ad^	824.30 ± 13.25 ^aj^	55.26 ± 0.01 ^aq^	698 ± 0.01 ^ax^	0.11 ± 0.00 ^aad^
Chestnut	Bragança	Vinhais	71.80 ± 0.12 ^d^	−3.22 ± 0.39 ^k^	19.84 ± 0.64 ^r^	117.20 ± 0.76 ^x^	108.68 ± 0.06 ^af^	1055 ± 0.00 ^ak^	73.39 ± 0.01 ^ar^	1418 ± 1.00 ^ay^	0.08 ± 0.00 ^aae^
Chestnut	Braga	Vila Verde	75.29 ± 0.13 ^e^	−3.72 ± 0.43 ^l^	13.95 ± 0.28 ^s^	121.38 ± 0.53 ^y^	69.63 ± 0.04 ^ag^	697.50 ± 0.26 ^al^	67.88 ± 0.01 ^as^	781 ± 0.55 ^az^	0.09 ± 0.00 ^aaf^
*Heather*	Coimbra	Vila Nova de Ceira	67.05 ± 0.11 ^f^	−1.93 ± 1.64 ^m^	35.71 ± 0.28 ^t^	106.67 ± 1.67 ^z^	332.69 ± 0.00 ^ah^	2112.87 ± 0.32 ^am^	83.75 ± 0.01 ^at^	1380 ± 0.50 ^aaa^	0.07 ± 0.00 ^aag^

Different letters (superscripts) in each column represent statistically significant differences at the confidence level *p* < 0.05. Superscripts have been inserted according to the hierarchy in lettering of the alphabet. Standard deviation values of EC_50_ were below 10^−4^. Therefore, these were considered as zeros. Results reported are the average ± standard deviation values of three replicates (*n* = 3).

**Table 4:** Semi-quantitative data (μg/kg) of Portuguese honeys according to botanical origin assuming a response factor equal to 1 for all the isolated compounds.

RT	Compounds (μg/kg)	RI_exp_	RI_lit_	Eucalyptus			Chestnut			Heather		
				Qualification	Avg	SD	Qualification	Avg	SD	Qualification	Avg	SD
6.41	2,3-Butanedione	<800	<800	ni	ni ^a^	ni	ni	ni ^a^	ni	83	31.40 ^b^	3.01
6.97	Acetic acid ethyl ester	<800	<800	ni	ni ^c^	ni	ni	ni ^c^	ni	91	148.94 ^d^	4.09
7.73	4-methyl-1,3-Pentadiene	<800	<800	ni	ni ^e^	ni	ni	ni ^e^	ni	94	26.31 ^f^	1.47
8.18	3-methyl-Butanal	<800	<800	ni	ni ^g^	ni	ni	ni ^g^	ni	95	115.75 ^h^	1.05
8.44	2-methyl-Butanal	<800	<800	ni	ni ^i^	ni	ni	ni ^i^	ni	89	141.01 ^j^	2.43
9.32	Heptane	<800	<800	93	4052.51 ^k^	1117.25	92	3842.64 ^k^	45.5	91	5930.61 ^l^	172.24
9.51	2,5-dimethyl-Furan	<800	<800	91	31.82 ^m^	9.18	ni	ni ^n^	ni	87	27.9 ^m^	1.91
9.85	2-Butanone	<800	<800	85	210.17 ^o^	229.79	ni	ni ^p^	ni	ni	ni ^p^	ni
10.08	2,4,5-trimethyl-1,3-Dioxolane	<800	<800	ni	ni ^q^	ni	86	32.70 ^r^	6.13	ni	ni ^q^	ni
10.60	2-methyl-2-Butenal	<800	<800	ni	ni ^s^	ni	94	95.98 ^t^	12.45	86	33.17 ^u^	5.62
10.77	dimethyl-Disulphide	<800	<800	95	23.34 ^v^	16.31	97	26.29 ^v^	7.51	96	21.68 ^v^	0.65
11.40	methyl-Benzene	<800	<800	ni	ni ^w^	ni	ni	ni ^w^	ni	95	99.61 ^x^	2.04
11.87	1-Octene	<800	<800	ni	ni ^y^	ni	ni	ni ^y^	ni	93	29.87 ^z^	0.49
11.94	2,3-Butanediol	<800	<800	ni	ni ^aa^	ni	83	110.53 ^ab^	34.79	ni	ni ^aa^	ni
12.10	Octane	800	800	95	956.00 ^ac^	592.93	93	507.77 ^ac^	228.98	95	637.85 ^ac^	32.33
13.08	2-Furancarboxaldehyde	826	835	ni	ni ^ad^	ni	95	94.21 ^ae^	43.23	95	1382.73 ^af^	55.01
13.37	2-methyl-Butanoic acid ethyl ester	837	846	ni	ni ^ag^	ni	ni	ni ^ag^	ni	92	44.05 ^ah^	8.35
13.46	3-methyl-Butanoic acid ethyl ester	841	839	ni	ni ^ai^	ni	80	36.56 ^aj^	2.53	ni	ni ^ai^	ni
13.96	ethyl-Benzene	860	862	ni	ni ^ak^	ni	85	12.58 ^al^	1.74	ni	ni ^ak^	ni
14.11	methoxy-phenyl-Oxime	865	-	83	10.70 ^am^	1.52	ni	ni ^an^	ni	91	19.68 ^ao^	0.76
14.19	1,3-dimethyl-Benzene	869	873	94	28.82 ^ap^	11.38	88	25.43 ^ap^	5.160	ni	ni ^aq^	ni
14.20	1,4-dimethyl-Benzene	869	877	ni	ni ^ar^	ni	ni	ni ^ar^	ni	92	32.84 ^as^	2.20
14.63	Pentanoic acid, ethyl ester	885	904	ni	ni ^at^	ni	85	28.29 ^au^	2.85	ni	ni ^at^	ni
14.71	Nonane	888	900	94	55.31 ^av^	1.71	96	46.50 ^av^	13.30	95	57.93 ^av^	0.47
14.77	ethenyl-Benzene	891	895	ni	ni ^aw^	ni	97	80.64 ^ax^	7.11	ni	ni ^aw^	ni
14.78	Benzene	891	-	97	78.55 ^ay^	3.79	ni	ni ^az^	ni	ni	ni ^az^	ni
15.11	1-(2-furanyl)-Ethanone	904	914	ni	ni ^aaa^	ni	ni	ni ^aaa^	ni	84	40.27 ^aab^	2.25
15.88	2,6,6-trimethyl-Bicyclo[3.1.1]hept-2-ene (a-Pinene)	936	943	96	47.78 ^aac^	7.96	96	31.66 ^aac^	21.09	91	76.21 ^aad^	0.99
16.38	5-methyl-2-Furancarboxaldehyde	957	954	ni	ni ^aae^	ni	ni	ni ^aae^	ni	94	42.83 ^aaf^	1.11
16.59	Benzaldehyde	966	970	94	70.19 ^aag^	80.27	97	539.28 ^aag^	396.68	96	1785.8 ^aah^	91.91
16.76	6-methyl-5-Hepten-2-one	973	986	93	19.59 ^aai^	6.17	91	23.64 ^aai^	3.60	ni	ni ^aaj^	ni
16.92	1-Decene	980	991	ni	ni ^aak^	ni	96	54.92 ^aal^	5.23	96	72.63 ^aam^	4.68
16.92	dimethyl-Trisulfide	980	966	94	74.66 ^aan^	25.65	ni	ni ^aao^	ni	ni	ni ^aao^	ni
16.98	Hexanoic acid ethyl ester	982	996	94	77.08 ^aap^	29.85	97	139.23 ^aap^	84.84	ni	ni ^aaq^	ni
17.11	Decane	988	1000	96	34.57 ^aar^	13.39	94	38.79 ^aar^	11.22	96	44.86 ^aar^	2.83
17.23	Octanal	993	1001	90	39.69 ^aas^	20.67	93	66.38 ^aas^	3.16	95	190.56 ^aat^	42.33
17.57	2-methyl-5-(1-methylethyl)-1,3-Cyclohexadiene (a-Phellandrene)	1007	1003	88	7.15 ^aau^	0.70	ni	ni ^aav^	ni	ni	ni ^aav^	ni
17.65	3,7,7-trimethylbicyclo[4.1.0]Hept-3-ene (Delta 3-Carene)	1011	1011	92	19.53 ^aaw^	4.38	ni	ni ^aax^	ni	ni	ni ^aax^	ni
17.74	2-ethyl-1-Hexanol	1015	1029	90	125.49 ^aay^	61.04	88	57.92 ^aay^	18.99	90	103.57 ^aaz^	9.67
17.95	1-methyl-2-(1-methylethyl)-Benzene	1025	1021	95	93.47 ^aaaa^	1.72	ni	ni ^aaab^	ni	ni	ni ^aaab^	ni
18.08	1-Methyl-4-(prop-1-en-2-yl)cyclohex-1-ene (dl-Limonene)	1031	1031	98	79.07 ^aaac^	15.07	99	51.65 ^aaad^	7.96	99	64.90 ^aaac^	2.84
18.25	1,3,3-trimethyl-2-oxabicyclo[2.2.2]Octane (Eucalyptol)	1038	1033	96	16.45 ^aaae^	2.98	98	38.02 ^aaaf^	4.61	ni	ni ^aaag^	ni
18.26	2,2,6-trimethyl-Cyclohexanone	1039	1036	ni	ni ^aaah^	ni	96	44.36 ^aaai^	5.85	ni	ni ^aaah^	ni
18.44	Benzene acetaldehyde	1047	1044	89	68.08 ^aaaj^	47.10	94	181.39 ^aaak^	37.90	94	1290.93 ^aaal^	85.78
18.56	2-hydroxy-Benzaldehyde	1053	1057	ni	ni ^aaam^	ni	98	37.93 ^aaan^	0.30	ni	ni ^aaam^	ni
18.67	1,4-Cyclohexadiene	1058	-	92	30.03 ^aaao^	3.52	ni	ni ^aaap^	ni	ni	ni ^aaap^	ni
18.74	alpha.-methyl-Benzenemethanol	1060	1066	ni	ni ^aaaq^	ni	94	28.30 ^aaar^	1.55	ni	ni ^aaaq^	ni
18.95	2-[(2S,5R)-5-ethenyl-5-methyloxolan-2-yl]propan-2-ol (cis-Linalool oxide)	1070	1074	91	165.75 ^aaas^	56.60	ni	ni ^aaat^	ni	91	1716.38 ^aaau^	121.07
19.16	Heptanoic acid, ethyl ester	1080	1083	ni	ni ^aaav^	ni	94	55.23 ^aaaw^	6.26	ni	ni ^aaav^	ni
19.30	2-[(2S,5S)-5-ethenyl-5-methyloxolan-2-yl]propan-2-ol (trans-Linalool oxide)	1086	1097	87	122.31 ^aaax^	59.09	89	99,2 ^aaax^	8.33	91	425.57 ^aaay^	29.37
19.38	(3R)-3,7-dimethylocta-1,6-dien-3-ol (Linalool L)	1090	1098	96	302.94 ^aaaz^	112.96	95	101,76 ^aaaaa^	30.33	ni	ni ^aaaab^	ni
19.47	Nonanal	1094	1102	ni	ni ^aaaac^	ni	82	257.63 ^aaaad^	5.24	ni	ni ^aaaac^	ni
19.48	3,7-Dimethyl-1,5,7-octatrien-3-ol (Hotrienol)	1094	1108	ni	ni ^aaaae^	ni	ni	ni ^aaaae^	ni	86	2085.89 ^aaaaf^	9.02
19.95	Phenylethylalcohol	1117	1121	ni	ni ^aaaag^	ni	ni	39.32 ^aaaah^	1.65	95	189.54 ^aaaai^	17.28
20.13	2,6-Dimethyl-1,3,5,7-octatetraene, (E,E-)	1126	1137	ni	ni ^aaaaj^	ni	ni	ni ^aaaaj^	ni	94	31.15 ^aaaak^	7.31
20.19	3,5,5-trimethyl-2-Cyclohexen-1-one (a-Isophorone)	1129	1120	ni	ni ^aaaal^	ni	89	35.26 ^aaaam^	0.77	91	475.51 ^aaaan^	26.94
20.41	2-Furanacetaldehyde,5-ethenyltetrahydro-α,5-dimethyl- (Lilac aldehyde B)	1139	1154	ni	ni ^aaaao^	ni	ni	ni ^aaaao^	ni	80	51.82 ^aaaap^	0.36
20.54	2,6,6-Trimethyl-2-cyclohexene-1,4-dione (4-Ketoisophorone)	1146	1143	96	91.70 ^aaaaq^	26.45	95	37.32 ^aaaar^	3.73	95	524.9 ^aaaas^	28.02
20.68	2-Hydroxy-3,5,5-trimethyl-cyclohex-2-enone (2-hydroxy-Isophorone)	1152	1150	92	83.63 ^aaaat^	49.51	92	22.79 ^aaaat^	1.55	95	91.18 ^aaaau^	5.43
20.76	1-ethenyl-4-methoxy-Benzene	1156	1155	ni	ni ^aaaav^	ni	ni	ni ^aaaav^	ni	96	157.75 ^aaaaw^	2.74
20.78	1-Nonanol	1157	1156	89	70.43 ^aaaax^	36.21	89	160.79 ^aaaax^	173.75	ni	ni ^aaaay^	ni
21.06	Benzoic acid ethyl ester	1171	1170	ni	ni ^aaaaz^	ni	94	81.80 ^aaaaaa^	34.23	95	419.62 ^aaaaab^	29.55
21.20	Octanoic acid ethyl ester	1178	1178	96	290.21 ^aaaaac^	77.59	95	246 ^aaaaac^	15.74	93	250.95 ^aaaaac^	5.76
21.37	endo-1,7,7-Trimethyl- bicyclo[2.2.1]heptan-2-ol (Borneol)	1186	1177	ni	ni ^aaaaad^	ni	90	49.46 ^aaaaae^	1.80	ni	ni ^aaaaaf^	ni
21.55	Decanal	1195	1195	90	40.26 ^aaaaag^	11.19	91	65.17 ^aaaaah^	8.73	91	72.10 ^aaaaah^	13.40
21.66	1-methyl-4-(propan-2-ylidene)cyclohex-1-ene (a-Terpinolene)	1200	-	ni	ni ^aaaaai^	ni	ni	ni ^aaaaai^	ni	92	29.21 ^aaaaaj^	3.27
21.82	2,6,6-trimethyl-1,3-Cyclohexadiene-1-carboxaldehyde (Safranal)	1209	1207	ni	ni ^aaaaak^	ni	94	19.99 ^aaaaal^	2.36	97	73.67 ^aaaaam^	1.32
21.82	Naphthalene	1209	1209	ni	ni ^aaaaan^	ni	87	11.57 ^aaaaao^	1.12	ni	ni ^aaaaan^	ni
22.28	3-phenyl-Furan	1233	1225	ni	ni ^aaaaap^	ni	89	50.53 ^aaaaaq^	55.07	93	216.75 ^aaaaar^	5.19
22.38	Benzeneacetic acid ethyl ester	1238	1244	91	57.99 ^aaaaas^	39.40	90	75.37 ^aaaaas^	4.63	91	148.17 ^aaaaat^	12.96
22.63	Benzothiazole	1251	1234	ni	ni ^aaaaau^	ni	ni	ni ^aaaaau^	ni	94	22.05 ^aaaaav^	1.07
22.92	4-methoxy-Benzaldehyde	1266	1252	ni	ni ^aaaaaw^	ni	ni	ni ^aaaaaw^	ni	97	208.33 ^aaaaax^	129.67
23.10	Nonanoic acid ethyl ester	1276	1294	98	278.88 ^aaaaay^	103.52	98	286.19 ^aaaaay^	115.41	98	212.78 ^aaaaay^	0.86
23.19	5-methyl-2-(1-methylethyl)-Phenol	1281	1292	ni	ni ^aaaaaz^	ni	ni	ni ^aaaaaz^	ni	93	95.02 ^aaaaaaa^	8.83
23.25	Tridecane	1284	1300	87	16.22 ^aaaaaab^	0.17	87	12.91 ^aaaaaab^	2.38	ni	ni ^aaaaaac^	ni
23.31	2-Undecanol	1287	1294	90	78.95 ^aaaaaad^	33.41	ni	ni ^aaaaaae^	ni	ni	ni ^aaaaaae^	ni
23.83	3,4,5-trimethyl-Phenol	1315	1320	ni	ni ^aaaaaaf^	ni	ni	ni ^aaaaaaf^	ni	95	163.68 ^aaaaaag^	12.63
23.86	1-(2-aminophenyl)-Ethanone	1317	1310	ni	ni ^aaaaaah^	ni	96	72.11 ^aaaaaai^	5.00	ni	ni ^aaaaaah^	ni
23.88	1-(6-methyl-3-pyridinyl)-Ethanone	1318	-	ni	ni ^aaaaaaj^	ni	91	17.33 ^aaaaaak^	1.45	ni	ni ^aaaaaaj^	ni
24.40	Benzenepropanoic acid ethyl ester	1347	1355	93	13.63 ^aaaaaal^	3.10	98	14.26 ^aaaaaal^	0.98	ni	ni ^aaaaaam^	ni
24.88	1,2-dihydro-1,1,6-trimethyl-Naphthalene	1374	1359	ni	ni ^aaaaaan^	ni	ni	ni ^aaaaaan^	ni	95	280.16 ^aaaaaao^	4.29
24.88	Decanoic acid ethyl ester	1374	1380	98	97.51 ^aaaaaap^	10.90	97	114.86 ^aaaaaap^	48.40	ni	ni ^aaaaaaq^	ni
25.09	(E)-1-(2,6,6-trimethyl-1-cyclohexa-1,3-dienyl)But-2-en-1-one (β-Damascenone)	1386	1385	96	81.07 ^aaaaaar^	38.54	94	17.74 ^aaaaaas^	1.95	97	181.51 ^aaaaaat^	7.83
25.25	2,4,4-trimethyl-3-carboxaldehyde-5-hydroxy-2,5-cyclohexadien-1-one	1395	-	94	56.22 ^aaaaaau^	58.04	ni	ni ^aaaaaav^	ni	ni	ni ^aaaaaav^	ni
26.06	3,5-dimethoxy-Benzaldehyde	1443	-	ni	ni ^aaaaaaw^	ni	ni	ni ^aaaaaaw^	ni	92	9.61 ^aaaaaax^	0.24
26.22	Benzoic acid, 4-methoxy-ethyl ester	1452	1468	ni	ni ^aaaaaaay^	ni	ni	ni ^aaaaaaay^	ni	92	23.84 ^aaaaaaaz^	2.39
26.45	(1aS,4aR,7aS,7bR)-1,1,7-trimethyl-4-methylidene-2,3,4a,5,6,7,7a,7b-octahydro-1aH-cyclopropa[e]azulene (Alloaromadendrene)	1466	1460	ni	ni ^aaaaaaab^	ni	97	16.41 ^aaaaaaac^	3.14	ni	ni ^aaaaaaab^	ni
26.53	2,6-bis (1,1-dimethylethyl)-2,5-Cyclohexadiene-1,4-dione	1471	1472	98	68.70 ^aaaaaaad^	37.35	99	81.61 ^aaaaaaad^	50.63	ni	ni ^aaaaaaae^	ni
26.83	5-methyl-2-phenyl-2-Hexenal	1489	1482	ni	ni ^aaaaaaaf^	ni	ni	ni ^aaaaaaaf^	ni	96	22.91 ^aaaaaaag^	1.13
26.71	Pentadecane	1481	1500	97	52.57 ^aaaaaaah^	19.60	97	36.10 ^aaaaaaah^	4.85	97	72.53 ^aaaaaaai^	1.47
27.41	1-bromo-Naphthalene	1524	-	97	10.07 ^aaaaaaaj^	3.79	97	7.06 ^aaaaaaaj^	1.06	94	51.29 ^aaaaaaak^	2.99
28.02	Naphthalene,1,2-dihydro-4,7-dimethyl-1-(1-methylethyl)-, (1S)(a-Calacorene)	1563	-	87	16.77 ^aaaaaaal^	2.07	ni	ni ^aaaaaaam^	ni	ni	ni ^aaaaaaam^	ni
28.15	Dodecanoic acid ethyl ester	1571	1590	99	51.86 ^aaaaaaan^	11.03	99	57.26 ^aaaaaaan^	19.33	ni	ni ^aaaaaaao^	ni
28.78	1,3,5-tris(1-methylethyl)-Benzene	1612	-	ni	ni ^aaaaaaap^	ni	ni	ni ^aaaaaaap^	ni	85	18.55 ^aaaaaaaq^	0.83
28.90	Bicyclo[5.3.0]decapentaene (Azulene)	1620	-	97	30.70 ^aaaaaaar^	10.83	ni	ni ^aaaaaaas^	ni	ni	ni ^aaaaaaas^	ni
29.78	Heptadecane	1679	1700	98	159.80 ^aaaaaaat^	75.10	ni	ni ^aaaaaaau^	ni	97	197.13 ^aaaaaaaw^	14.44
32.81	Eicosane	1876	2000	ni	ni ^aaaaaaaw^	ni	ni	ni ^aaaaaaaw^	ni	98	243.75 ^aaaaaaax^	5.40
34.48	Hexadecanoic acid ethyl ester	1966	1975	ni	ni ^aaaaaaaay^	ni	97	31.91 ^aaaaaaaaz^	2.60	ni	ni ^aaaaaaay^	ni
	Sum of Volatiles (μg/kg)				8488.22^7 az^	3180.80		8448.52^7 az^	1626.72		21257.17^7 aza^	1051.57

RT: retention time, RI_exp_: experimental retention indices values based on the calculations using the standard mixture of alkanes. RI_lit_: Retention indices of the identified compounds according to literature data cited in Wiley 7 NIST MS library. Qualification: Percentage accuracy of volatile compounds identified using Wiley 7 NIST MS data Different letters (superscripts) in each row indicate statistically significant differences at the confidence level *p* < 0.05. Superscripts have been inserted according to the hierarchy in lettering of the alphabet. ni: not identified; were treated as zeros for statistical analysis. Results reported are the average ± standard deviations values of two independent replicates (*n* = 2).
